# Expansion of Single Cell Transcriptomics Data of SARS-CoV Infection in Human Bronchial Epithelial Cells to COVID-19

**DOI:** 10.1186/s12575-020-00127-3

**Published:** 2020-07-23

**Authors:** Reza Zolfaghari Emameh, Hassan Nosrati, Mahyar Eftekhari, Reza Falak, Majid Khoshmirsafa

**Affiliations:** 1 Department of Energy and Environmental Biotechnology, National Institute of Genetic Engineering and Biotechnology (NIGEB), 14965/161, Tehran, Iran; 2 Department of Materials Engineering, Tarbiat Modares University, Tehran, Iran; 3 Immunology Research Center, Iran University of Medical Sciences, Tehran, Iran; 4 Immunology Department, School of Medicine, Iran University of Medical Sciences, Tehran, Iran

**Keywords:** Transcriptomics, Bronchial epithelial cells, Immunological regulations, Signaling pathways, Cytokine storm, Gene ontology, Reactome pathways, Severe acute respiratory syndrome, SARS-CoV-2, COVID-19

## Abstract

**Background:**

Severe acute respiratory syndrome coronavirus 2 (SARS-CoV-2) is the causative agent of coronavirus disease 19 (COVID-19) that was emerged as a new member of coronaviruses since December 2019 in Wuhan, China and then after was spread in all continentals. Since SARS-CoV-2 has shown about 77.5% similarity to SARS-CoV, the transcriptome and immunological regulations of SARS-CoV-2 was expected to have high percentage of overlap with SARS-CoV.

**Results:**

In this study, we applied the single cell transcriptomics data of human bronchial epithelial cells (2B4 cell line) infected with SARS-CoV, which was annotated in the Expression Atlas database to expand this data to COVID-19. In addition, we employed system biology methods including gene ontology (GO) and Reactome pathway analyses to define functional genes and pathways in the infected cells with SARS-CoV. The transcriptomics analysis on the Expression Atlas database revealed that most genes from infected 2B4 cell line with SARS-CoV were downregulated leading to immune system hyperactivation, induction of signaling pathways, and consequently a cytokine storm. In addition, GO:0016192 (vesicle-mediated transport), GO:0006886 (intracellular protein transport), and GO:0006888 (ER to Golgi vesicle-mediated transport) were shown as top three GOs in the ontology network of infected cells with SARS-CoV. Meanwhile, R-HAS-6807070 (phosphatase and tensin homolog or PTEN regulation) showed the highest association with other Reactome pathways in the network of infected cells with SARS-CoV. PTEN plays a critical role in the activation of dendritic cells, B- and T-cells, and secretion of proinflammatory cytokines, which cooperates with downregulated genes in the promotion of cytokine storm in the COVID-19 patients.

**Conclusions:**

Based on the high similarity percentage of the transcriptome of SARS-CoV with SARS-CoV-2, the data of immunological regulations, signaling pathways, and proinflammatory cytokines in SARS-CoV infection can be expanded to COVID-19 to have a valid platform for future pharmaceutical and vaccine studies.

## Background

The transcriptome of living organisms including RNA transcripts is studied by transcriptomics assays [1]. The coding genes of organisms are present in the DNA, which are transcribed to mRNA by transcription. Two major techniques have widespread applications in the transcriptomics analysis of cell mRNA pool: the first one is microarray which is used for detection of a labeled mRNA or surrogate marker by an immobilized probe [2] and the second one is RNA-Seq which is a high-throughput sequencing technique, capable to differentiate various types of RNA including miRNA, tRNA, and rRNA [3]. The outputs of RNA-Seq analysis can be validated by quantitative reverse transcription PCR (RT-qPCR) as a complementary method in transcriptomics [4]. Recently, the transcriptome of infected human tissue samples was detected by analyzing the site of infection [5] to elucidate the response of patients to external pathogens and understand the molecular regulatory mechanisms of infections [6]. Two models, including cell lines for in vitro and animal models for in vivo assays are commonly considered for transcriptomics analysis of human responses to infectious agents [7]. In vitro and in vivo cell lines/animals interactions with pathogens and their host-specific responses to the pathogens can be evaluated by transcriptomics techniques. The interactions of the infected cell lines with immune system cellular components can be through recognition with specific T cells and natural killer (NK) cells as the crucial factors of the adaptive and innate immunity and reflected in the transcriptional responses [8–10]. In addition, the patterns of gene expression as a biomarker can be evaluated by transcriptomics techniques to discriminate the different stages of the infections and may influence physiological pathways.

Previously, transcriptome analyses of host-pathogen interactions were conducted on the viral infections including herpesvirus [11], cytomegalovirus [12, 13], influenza virus [14], human respiratory syncytial virus [15], measles virus [16], rubella virus [17], dengue virus [18], rabies virus [17], ebolavirus [19, 20], and human immunodeficiency virus (HIV) [21]. Moreover, studding the transcriptome of Calu-3 cell line (human lung cancer cell line) after infection with severe acute respiratory syndrome coronavirus (SARS-CoV) revealed that recognition receptors and the interleukin 17 (IL-17) pathway were activated [22]. In addition, another analysis was performed on the transcriptome of Calu-3 cell line following Middle East respiratory syndrome coronavirus (MERS-CoV) infection to identify suitable inhibitory and antiviral compounds [23].

Since December 2019, SARS-CoV-2 was emerged as a novel member of coronaviruses in Wuhan, China [24] and soon after spread around the world [25, 26]. In a study in 2005 that was performed on the prophylactic and therapeutic supremacies of chloroquine, as an anti-malaria agent, on primate cells infected with SARS-CoV, it was defined that chloroquine increases the endosomal pH and intervene with glycosylation of angiotensin-converting enzyme 2 (ACE2) as the receptor of SARS-CoV on the alveolar cell surface [27]. Although chloroquine and hydroxychloroquine sulfate, as the anti-malaria agents, were prescribed for treatment of coronavirus disease 2019 (COVID-19) at the beginning of pandemic [28], the ocular toxicity and cardiotoxicity of chloroquine and hydroxychloroquine sulfate should be considered [29, 30].

In a study performed by Wang C et al. in 2020, it was defined that the spike (S) protein of Wuhan strain of SARS-CoV-2 contains 1273 amino acid residues and the S protein of Urbani strain of SARS-CoV includes 1255 amino acid residues that showed about 77.5% similarity between the amino acid residues of S protein of both viruses. The study on the receptor binding domain (RBD) of S protein from both SARS-CoV and SARS-CoV-2 by enzyme linked immunosorbent assay (ELISA) and human 47D11 antibody revealed similar affinities of the human 47D11 antibody to ACE2 receptor of host cells through S1_B_ domain of both SARS-CoV and SARS-CoV-2. In this study, 25 human and non-human cell lines from various organs and tissues were inoculated with SARS-CoV and SARS-CoV-2 and finally similar tropism of both viruses to ACE2 receptor was confirmed; meanwhile, they declared that the observed differences could be due to different infection capacity in the target organs such lungs, kidneys, and brain, resulting different cytopathic-effects in the tested cell lines [31–33].

Since the recent emergence of SARS-CoV-2 and considering its high transmission rate [34] which have affected all aspects of human life including health and economy [35, 36], it would be an urgent matter to combat against it and find an effective vaccine and/or potential therapeutics to treat COVID-19. Within this context, we try to expand the transcriptomics data of the interaction of lung cells with SARS-CoV to infection of bronchoalveolar cells with SARS-CoV-2. Due to the high percentage of similarity between SARS-CoV and SARS-CoV-2, this approach could be a potential solution in the pharmaceutical studies to explore an influential compound to treat COVID-19 efficiently. In this study and through the in silico and system biology approaches, we checked the transcriptomics data of infection of lung bronchial cells with SARS-CoV and believe that the results can be potentially expanded to infection of host lung bronchial epithelial cells with SARS-CoV-2 in COVID-19 patients.

## Methods

### mRNA Microarray Assay

The microarray assay on the mRNAs from 2B4 cell line (human bronchial epithelial cell) after infection with SARS-CoV was performed using Affymetrix GeneChip Human Genome U133 Plus 2.0 [HG-U133_Plus_2] by Yoshikawa et al. in 2010 [37]. The transcriptomics data of the infected 2B4 cell line was recorded for three different time durations including 12 h, 24 h, and 48 h post infection with SARS-CoV and the analysis was adjusted on *p*-value< 0.05 and Log_2_-fold change 1.0. This transcription profile was annotated in the Expression Atlas (https://www.ebi.ac.uk/gxa/home) hosted by EMBL-EBI database [38].

### Gene Ontology (GO) Analysis

The GO analysis was conducted to identify the functionality of altered 2B4 cell line genes after infection with SARS-CoV. Through a computational approach, the function of altered genes was predicted by both human- and machine-readable data and the obtained results were filtered by Fisher’s exact test [39], False Discovery Rate (FDR)<1.0. This statistical significance removed the proportion of false positives among all upregulated and downregulated genes from 2B4 cell line genes after infection with SARS-CoV.

### Reactome Pathways Analysis

The Reactome pathways analysis was conducted to explore the biochemical and physiological pathways in the infected 2B4 cell line with SARS-CoV. The same as GO analysis, Fisher’s exact test [39] and FDR<1.0 was employed to statistically identify the activated Reactome pathways in the 2B4 cell line genes after infection with SARS-CoV.

## Results

### mRNA Microarray Assay

**Fig. 1:**
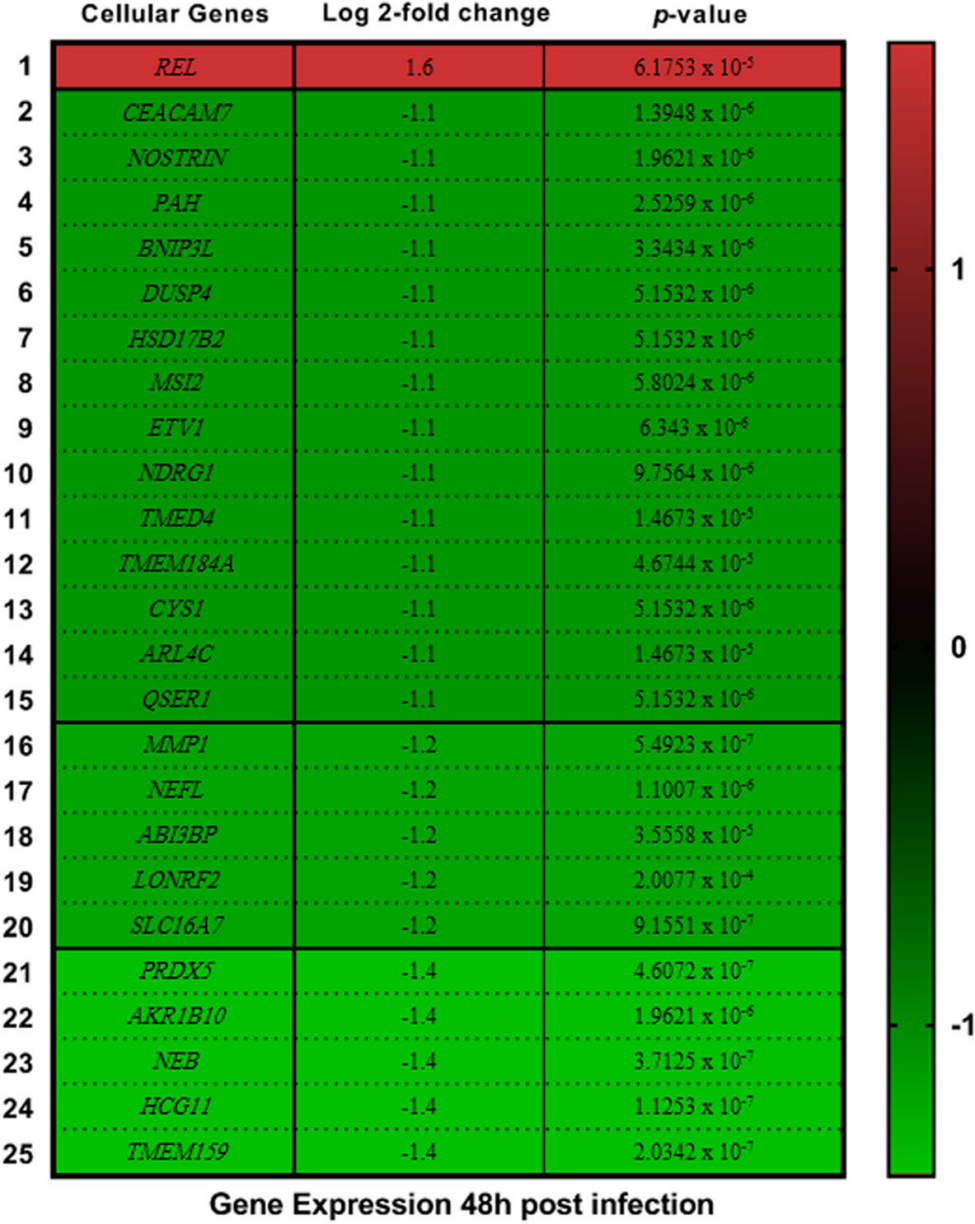
Expression variations of cellular genes after SARS-CoV infection. The 2B4 cell line was infected with SARS-CoV and following 48 h incubation the gene expression was analyzed by microarray method. Most of the affected genes showed slight downregulation. Colors indication: dark red for low level upregulation, dark green for low level downregulation, and light green for high level downregulation. The analysis was adjusted on *p*-value< 0.05 and Log2-fold change 1.0

The microarray mRNA analysis of the annotated data in the Expression Atlas database [38] revealed that no significant gene regulation occurs in the host cells within 24 h. All genes related to the interaction of 2B4 cell line and SARS-CoV were expressed after 48 h, in which most of the affected genes were downregulated, while just *REL* (c-Rel proto-oncogene) gene was upregulated (Fig. 1).

**Fig. 2:**
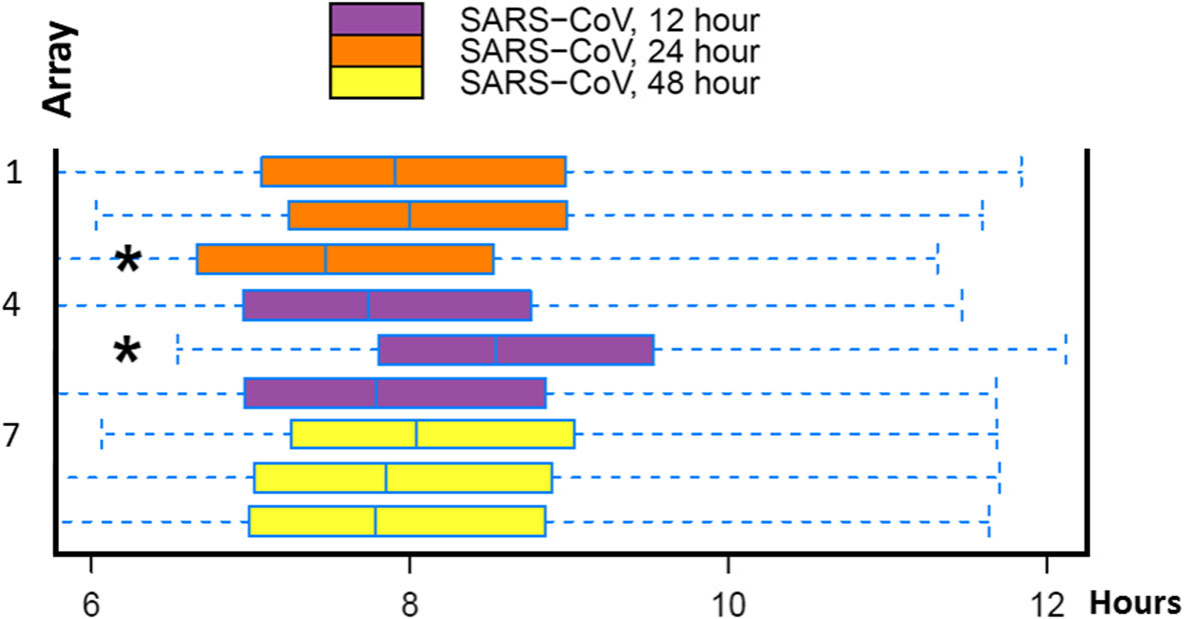
Boxplots show the array intensity distributions of SARS-CoV genes. The outlier computation method between the distribution of the pooled data and each array’s distribution derived from Kolmogorov-Smirnov statistic *K*_*a*_. Asterisks indicate inaccurate results in the experiments. Purple bars show the expression level of SARS-CoV genes after 12 h, orange bars show the expression level of SARS-CoV genes after 24 h, and yellow bars show the expression level of SARS-CoV genes after 48 h. All three experiments demonstrate that SARS-CoV genes are expressed between 7 h and 9 h post infection of alveolar cells

The microarray analysis on the expression of SARS-CoV genes in all three different durations (12 h, 24 h, and 48 h) of interaction with 2B4 cell line demonstrated almost similar expression level of SARS-CoV genes between 7 h to 9 h after infection of 2B4 cell line (Fig. 2).

### Gene Ontology (GO) Analysis

**Fig. 3:**
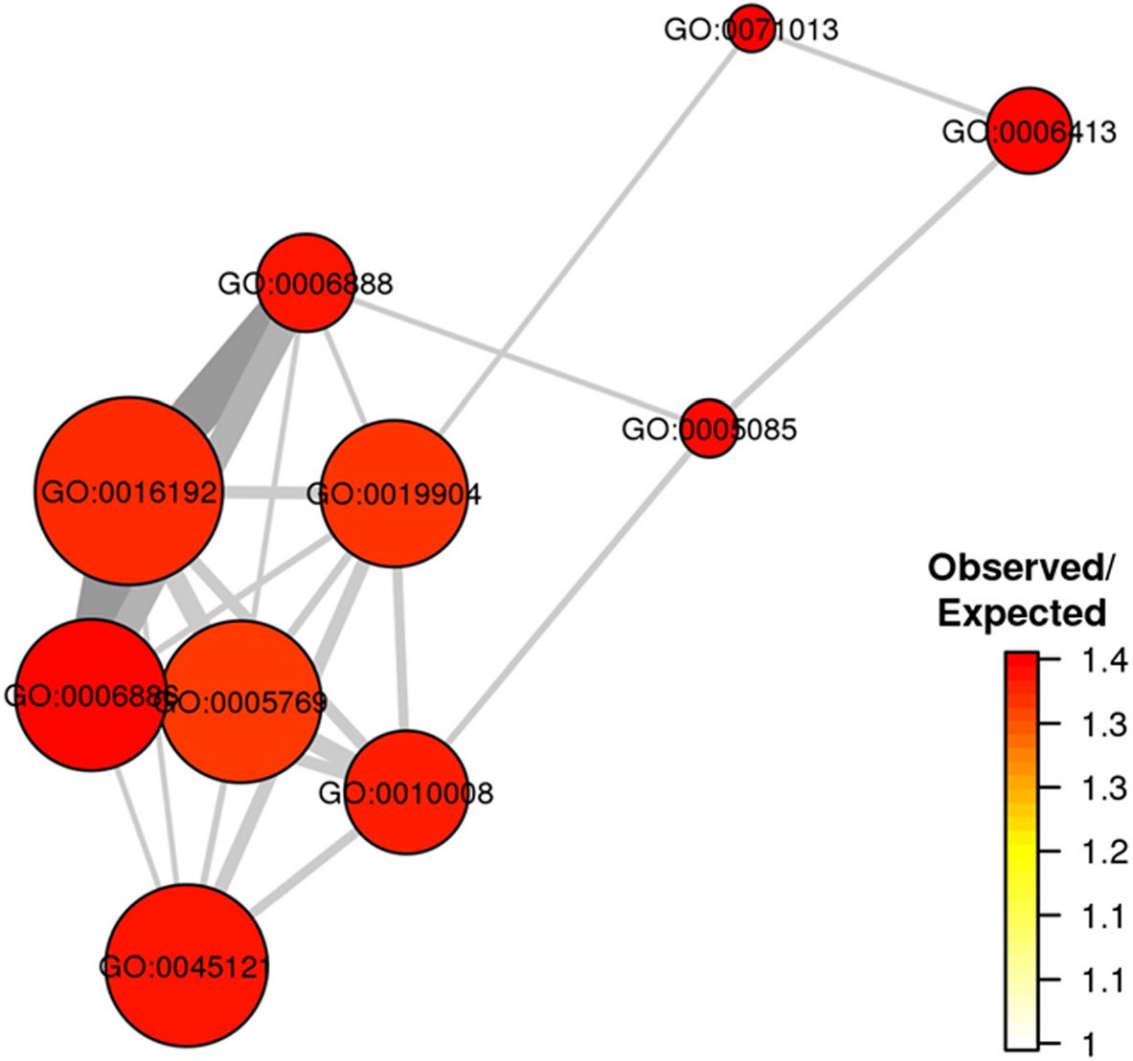
Enrichment of top ten gene ontology (GO) after infection of 2B4 cell line with SARS-CoV. The GO analysis shows the top ten cellular genes are regulated after SARS-CoV infection

In this analysis, the top ten GO as a network were visualized that their gene products were attributed to some vital cellular functions of the infected 2B4 cell line with SARS-CoV. Three out of ten GO including GO:0016192 (vesicle-mediated transport), GO:0006886 (intracellular protein transport), and GO:0006888 (ER to Golgi vesicle-mediated transport) displayed the highest association in the GO network after infection of 2B4 cell line with SARS-CoV (Fig. 3) (Table 1).

**Table 1:** The top ten GO analysis and Reactome pathways after infection of 2B4 cell line with SARS-CoV

Top 10	GO analysis	Reactome enrichment pathways
**1**	GO:0005769 (early endosome)	R-HAS-9612973 (autophagy)
**2**	GO:0019904 (protein domain specific binding)	R-HAS-6807070 (PTEN regulation)
**3**	GO:0016192 (vesicle-mediated transport)	R-HAS-5607764 (CLEC7A or Dectin-1 signaling)
**4**	GO:0010008 (endosome membrane)	R-HAS-163200 (respiratory electron transport, ATP synthesis by chemiosmotic coupling, and heat production by uncoupling proteins)
**5**	GO:0045121 (membrane raft)	R-HAS-4086400 (PCP/CE pathway)
**6**	GO:0006888 (ER to Golgi vesicle-mediated transport)	R-HAS-428157 (sphingolipid metabolism)
**7**	GO:0005085 (guanyl-nucleotide exchange factor activity)	R-HAS-156827 (L13a-mediated translational silencing of Ceruloplasmin expression)
**8**	GO:0006413 (translational initiation)	R-HAS-72706 (GTP hydrolysis and joining of the 60S ribosomal subunit)
**9**	GO:0006886 (intracellular protein transport)	R-HAS-179419 (APC:Cdc20-mediated degradation of cell cycle proteins prior to satisfaction of the cell cycle checkpoint)
**10**	GO:0071013 (catalytic step 2 spliceosome)	R-HAS-72613 (eukaryotic translation initiation)

### Reactome Pathways Analysis

**Fig. 4:**
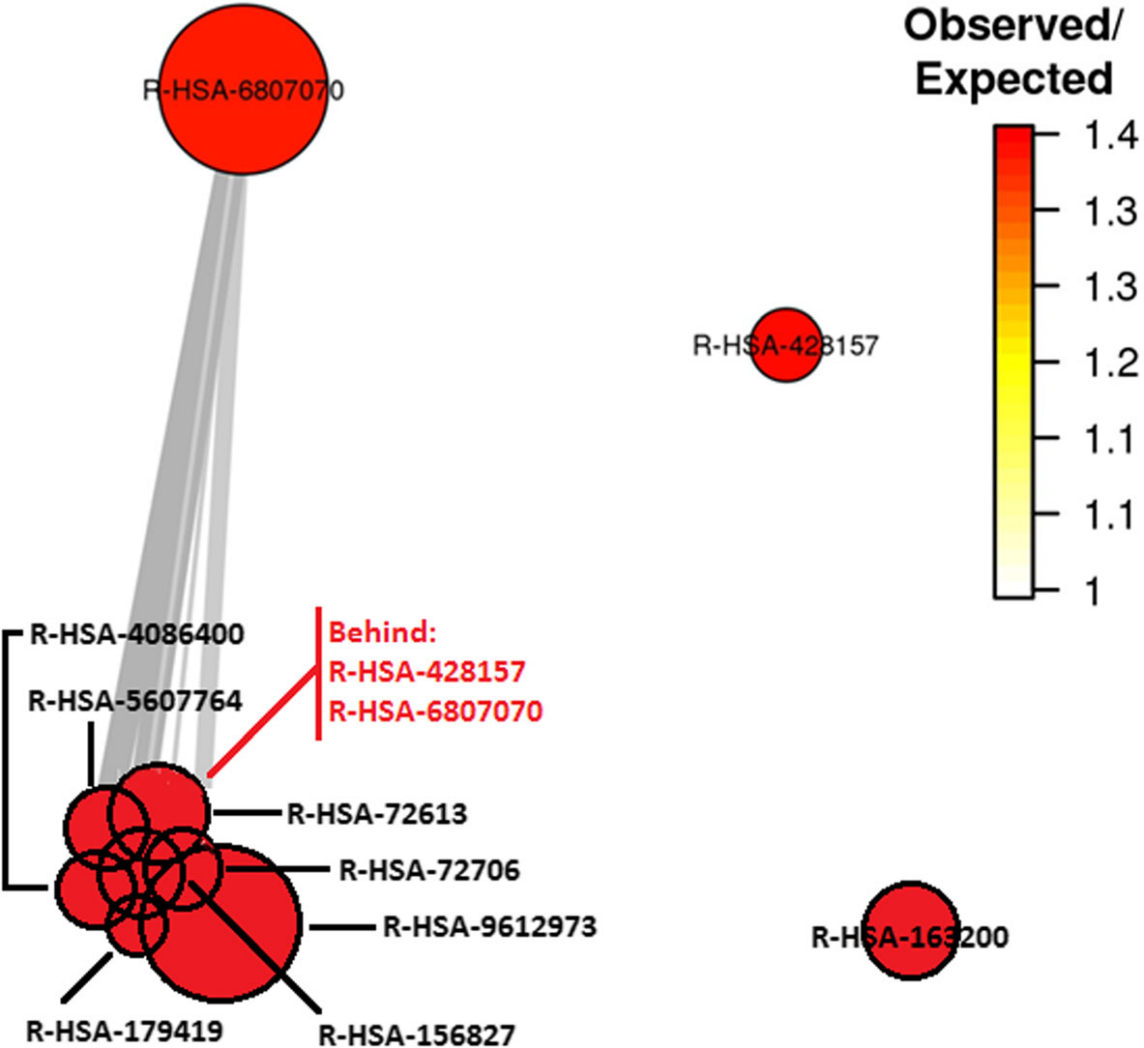
Enrichment of top ten Reactome pathways after infection of 2B4 cell line with SARS-CoV. The Reactome pathways show the top ten cellular pathways and networks are enriched after SARS-CoV infection

In this analysis, the top ten Reactome pathways as a network were visualized that showed a significant attribution in the biochemical and physiological pathways after infection of 2B4 cell line with SARS-CoV. One out of ten Reactome pathways including R-HAS-6807070 (phosphatase and tensin homolog or PTEN regulation) showed the highest association with other Reactome pathways in the network of infected 2B4 cell line with SARS-CoV (Fig. 4) (Table 1).

## Discussion

The study of literature demonstrated that the blood transcriptome of the infected host with SARS-CoV could be mined by reverse vaccinology and system vaccinology technologies and consequently a potential vaccine candidate could be designed through the production of recombinant proteins against COVID-19 [40]. The exploration of pathogenesis mechanisms of SARS-CoV infection can provide more detailed information to design drugs against COVID-19 through the regulation of immune response [41]. The transcriptomic gene expression profiles of Yellow Fever vaccine 17D (YF-17D) were analyzed by a GO system named Vaccine Investigation Ontology (VIO) to identify the association of various variables in the vaccinated population. Hence, not only GO analysis has a potential application in the microarray assay after infection and/or vaccination to detect the profiles of gene expression, but also the Reactome analysis pathway tools are employed to explore the enriched pathways after infection and/or vaccination [42]. Therefore, the *p-*value based on FDR < 0.05 as the significance cut-offs was applied in the GO analysis and Reactome pathway enrichment pathways in the evaluation of transcriptomic gene expression profiles by microarray assay in SARS-CoV infection, which has a potential to be expanded to CIVID-19.

In a study performed by Mizutani et al. in 2005, the in vitro and in vivo signaling pathways in SARS-CoV infection were illustrated [43]. In response to stressors, p38 mitogen-activated protein kinase (MAPK) is expressed so two isoforms of MAPK including p38α and p38β are expressed in all tissues, while the expression of other two isoforms including p38γ and p38δ is tissue-specific so the inhibition of p38α and/or p38β MAPKs in SARS-CoV infection would be occurred by SB203580 inhibitor. In addition, the interaction between MAPK kinase 6 (MKK6) and protein kinase R (PKR) activates p38 MAPK [44]. The activation of PKR is done by MKK6 through double-stranded RNA, while the activation of MKK6 is done by PKR through poly(rI;rC). Apoptosis signal-regulatory kinase (ASK1), transforming growth factor (TGF)-β-activating kinase (TAK1), and MAPKKK4 were categorized as the upstream targets of p38MAPK [45], while MAP kinase-interacting kinase 1 (MNK1), mitogen and stress-activated protein kinase 1 (MSK1), and MAPK-activated protein kinase 2 and 3 (MAPKAPK 2 and 3) were known as the downstream targets of p38MAPK [46]. On the other hand, the 7a protein from SARS-CoV induces phosphorylation and apoptosis of p38 MAPK in 293 T cells [47], while the induction of p38 MAPK pathway induces actin reorganization in COS-1 cells leading to devoid of growth factors [48].

The microarray analysis on the infected 2B4 cell line with SARS-CoV was performed by Yoshikawa et al in 2010 [37]. The results of this study revealed that *REL* gene was upregulated. *REL* is a member of nuclear factor-κB (NF-κB) family of transcription factors. It was defined that the pathogenesis of SARS-CoV is associated with stimulated induction of proinflammatory cytokines by activation of at least five pathways including NF-κB, NF-AT, IRF-3, IRF-7, ATF-2/jun, and jun/fos (AP-1) [49]. Similarly in COVID-19, it can be expected that the activation of REL gene can lead to cytokine storm in the infected lung with SARS-CoV-2. Cytokine storm is an uncontrolled systemic inflammatory response that may lead to multi-organ failure and death in COVID-19.

Downregulation of peroxiredoxin 5 (*PRDX5*) gene levels by SARS-CoV infection in the 2B4 cell line reduces the intracellular hydrogen peroxide (H_2_O_2_) levels to minimize oxidative stress in the infected cells [50]. The expression of matrix metalloproteinase 1 (*MMP1*) gene was downregulated in SARS-CoV infection, while *MMP1* gene is overexpressed in influenza H7N9, which may lead to deposition of collagen in lungs and consequently gas exchange problem due to fibrosis [51, 52]. Therefore, it seems that the occurrence mechanism of acute respiratory distress syndrome (ARDS) in COVID-19 and influenza infection is initiated by two different mechanisms. Neurofilament triplet L protein (*NEFL*) gene can be downregulated in the SARS-CoV infection, while *NEFL* gene is upregulated in Zika virus infected cells [53]. Another downregulated gene in the SARS-CoV infection is nitric oxide synthase traffic inducer (*NOSTRIN*) so this downregulation can induce the secretion of some proinflammatory chemokines and cytokines including CCL2, CCL5, and IL-6 [54]. This is in controversy with the high secretion of IL-6 in COVID-19 which is an initiator mechanism for C-reactive protein (CRP) production and cytokine storm [55]. Another study showed that phenylalanine hydroxylase (*PAH*) gene was downregulated in SARS-CoV infection leading to activation of immune system through upregulation of T helper 1 (Th1) responses [56]. Notably, the cytokine storm is usually amplified by activation and secretion of cytokines from Th1 lymphocytes [57]. Lower expression of BCL2 Interacting Protein 3 like (*BNIP3L*) gene was experienced in SARS-CoV infection, while downregulation of this gene was associated with a reduction of NK cell memory and their response to cytomegalovirus infection [58]. Dual specificity protein phosphatase 4 (*DUSP4*) gene was downregulated in SARS-CoV infection with the association with the skew of Th1 toward Th2 immune response leading to susceptibility to viral infections and inflammation, however, may control the occurrence of cytokine storm by IL-6, IL-12 and tumor necrosis factor α (TNFα) as well as increasing the amount of prostaglandin PGE_2_ [59]. Another study showed that the coding gene for hydroxysteroid 17-beta dehydrogenase 2 (*HSD17B2*) was downregulated in SARS-CoV infection, which is a consequence of stimulation of IL-1, IL-6, and TNFα as well as induction of inflammation. On the other hand, the coding gene for Musashi-2 (*MSI2*) RNA-binding protein was downregulated in SARS-CoV infection. The loss of function of *MSI2* gene affects hematopoietic cells and consequently the development of leukocytes leading to suppression of the innate immune responses to infections [60, 61]. In SARS-CoV infection, the downregulation of *N-myc* downstream-regulated 1 (*NDRG1*) gene was led to induction of the proinflammatory signals and activation of a cytokine storm with C-C Motif Chemokine Ligand such as CCL2, CCL5, CCL23, CCL26, C-X-C Motif Chemokine Ligands such CXCL10, CXCL11, CXCL16, Vav Guanine Nucleotide Exchange Factor 3 (VAV3), signal transducer and activator of transcription 1 (STAT1), and SHC-transforming protein 3 (SHC3) [62]. In addition to aforementioned genes, the coding gene for transmembrane emp24 domain-containing protein 4 (*TMED4*) was downregulated in SARS-CoV infection. TMED4 can interact with interleukin-1 receptor-like 1 so the production of proinflammatory cytokines including IL-6 and IL-8 was induced [63]. Another downregulated gene in the SARS-CoV infection was transmembrane protein 184A (*TMEM184A*), which was necessary for heparin responses in endothelial cells and vascular smooth muscle cells with interruption effect on the anti-inflammatory responses of endothelial cells to heparin [64]. The reduction in the production level of taurine and induction of inflammatory responses was experienced by downregulation of nebulin (*NEB*) gene in SARS-CoV infection [65]. In addition, mitogen-activated protein kinase (MAPK) signaling pathway was activated after downregulation of *HCG11* gene leading to increased production of inflammatory cytokines [66]. Another downregulated gene was cysteine synthase (*CYS1*). Previous studies have shown that the presence of some amino acids such as cysteine are essential for the optimization of immune system and immunomodulatory properties including regulation of proinflammatory cytokines and T-cell proliferation, so the presence of cysteine-rich proteins in the diet can improve the function of immune system during the infections. *CYS1* downregulation can be led to the activation of NF-κB, stimulation of macrophages, and cytokine storm by over production of IL-6 and TNF-α [67, 68]. Moreover, an association was reported between the downregulation of glutamine and serine-rich protein 1 (*QSER1*) and bromodomain containing 1 (*BRD1*) genes, which consequently induce the infiltration of B-cells and activate humoral immune responses [69]. The results obtained from mRNA microarray assay [37] revealed that the most downregulated genes in the infected bronchial epithelial cells with SARS-CoV induce signaling pathways and interleukin-producing cells toward an overactivation of immune system leading to cytokine storm, which the mentioned outcomes can be expanded to COVID-19 as well.

GO analysis defined top three GOs including GO:0016192, GO:0006886, and GO:0006888, which are intermediate transportation forms for exocytosis of assembled SARS-CoV proteins by smooth-wall vesicles to plasma membrane [70], intracellular transportation of 3a protein from SARS-CoV with the significant role of YXXΦ motif [71], and cycling S protein through the endoplasmic reticulum (ER)-Golgi system [72]; respectively. Due to similar proteins of SARS-CoV and SARS-CoV-2, the results of GO analysis from SARS-CoV can be expanded to COVID-19 as well.

The Reactome pathway analysis revealed that PTEN homolog plays a crucial role in the SARS-CoV infection through activation of dendritic cells, production of hyperactive B-cells and uncontrolled T-cells, and secretion of proinflammatory cytokines including interferons (IFNs), TNF-α, IL-10, IL-4, and granulocyte monocyte-colony stimulating factor (GM-CSF) [73]. Therefore, similar to SARS-CoV infection, PTEN Reactome pathway can regulate several Reactome pathways and immune responses in COVID-19.

## Conclusions

Based on the high percentage of similarity of SARS-CoV and SARS-CoV-2, the results of many former studies on SARS-CoV can be expanded to SARS-CoV-2 to accelerate the pharmaceutical and vaccine explorations against COVID-19. Study on the single cell transcriptomics of infected bronchial epithelial cells with SARS-CoV demonstrates several immunity regulation factors and pathways that can be expanded to SARS-CoV-2 immunological pathogenesis and capable to lead a cytokine storm in the COVID-19 patients. As the overall conclusion, the transcriptomics data from SARS-CoV infection has the potential to be a golden pattern in the future pharmaceutical and vaccine studies against COVID-19.

## Data Availability

All data analyzed in this study were prepared from Expression Atlas database and were included in this article.

## Abbreviations

ACE2: Angiotensin-converting enzyme 2
ARDS: Acute respiratory distress syndrome
ASK1: Apoptosis signal-regulatory kinase
BNIP3L: BCL2 Interacting Protein 3 like
BRD1: Bromodomain containing 1
COVID-19: Coronavirus disease 2019
CRP: C-reactive protein
CYS1: Cysteine synthase
ELISA: Enzyme linked immunosorbent assay
ER: Endoplasmic reticulum
FDR: FalseDiscoveryRate
GM-CSF: Granulocyte monocyte-colony stimulating factor
GO: Gene ontology
HSD17B2: Hydroxysteroid 17-beta dehydrogenase 2
IFN: Interferon
IL: Interleukin
MAPK: Mitogen-activated protein kinase
MAPKAPK: MAPK-activated protein kinase
MERS-CoV: Middle East respiratory syndrome coronavirus
MKK6: MAPK kinase 6
MMP1: Matrix metalloproteinase 1
MNK1: MAP kinase-interacting kinase 1
MSI2: Musashi-2
MSK1: Mitogen and stress-activated protein kinase 1
NDRG1: N-myc downstream-regulated 1
NEB: Nebulin
NEFL: Neurofilament triplet L protein
NF-κB: Nuclear factor-κB
NK cell: Natural killer cell
NOSTRIN: Nitric oxide synthase traffic inducer
PAH: Phenylalanine hydroxylase
PKR: Protein kinase R
PRDX5: Peroxiredoxin 5
PTEN: Phosphatase and tensin
QSER1: Glutamine and serine-rich protein 1
RBD: Receptor binding domain
RT-qPCR: Quantitative reverse transcription PCR
S: Spike
SARS-CoV-2: Severe Acute Respiratory Syndrome Coronavirus 2
SHC3: SHC-transforming protein 3
STAT1: Signal transducer and activator of transcription 1
TAK1: Transforming growth factor (TGF)-β-activating kinase
Th: T helper
TMED4: Transmembrane emp24 domain-containing protein 4
TNFα: Necrosis factor α
VAV3: Vav Guanine Nucleotide Exchange Factor 3
